# 7 Tesla Magnetic Resonance Imaging to Detect Cortical Pathology in Multiple Sclerosis

**DOI:** 10.1371/journal.pone.0108863

**Published:** 2014-10-10

**Authors:** Bing Yao, Simon Hametner, Peter van Gelderen, Hellmuth Merkle, Christina Chen, Hans Lassmann, Jeff H. Duyn, Francesca Bagnato

**Affiliations:** 1 Advanced Magnetic Resonance Imaging Section, Laboratory of Functional and Molecular Imaging, National Institute of Neurological Disorders and Stroke, NIH, Bethesda, Maryland, United States of America; 2 Center for Neuroimaging Research, Kessler Foundation, West Orange, New Jersey, United States of America; 3 Center for Brain Research, Medical University, Vienna, Austria; 4 Neuroimmunology Branch, National Institute of Neurological Disorders and Stroke, NIH, Bethesda, Maryland, United States of America; 5 Department of Radiology and Radiological Science, Institute of Imaging Science, Vanderbilt University, Nashville, Tennessee, United States of America; University Medical Center Goettingen, Germany

## Abstract

**Background:**

Neocortical lesions (NLs) are an important pathological component of multiple sclerosis (MS), but their visualization by magnetic resonance imaging (MRI) remains challenging.

**Objectives:**

We aimed at assessing the sensitivity of multi echo gradient echo (ME-GRE) T_2_^*^-weighted MRI at 7.0 Tesla in depicting NLs compared to myelin and iron staining.

**Methods:**

Samples from two MS patients were imaged *post mortem* using a whole body 7T MRI scanner with a 24-channel receive-only array. Isotropic 200 micron resolution images with varying T_2_^*^ weighting were reconstructed from the ME-GRE data and converted into R_2_^*^ maps. Immunohistochemical staining for myelin (proteolipid protein, PLP) and diaminobenzidine-enhanced Turnbull blue staining for iron were performed.

**Results:**

Prospective and retrospective sensitivities of MRI for the detection of NLs were 48% and 67% respectively. We observed MRI maps detecting only a small portion of 20 subpial NLs extending over large cortical areas on PLP stainings. No MRI signal changes suggestive of iron accumulation in NLs were observed. Conversely, R_2_^*^ maps indicated iron loss in NLs, which was confirmed by histological quantification.

**Conclusions:**

High-resolution *post mortem* imaging using R_2_^*^ and magnitude maps permits detection of focal NLs. However, disclosing extensive subpial demyelination with MRI remains challenging.

## Introduction

Neocortical lesions (NLs) are an important pathological component of multiple sclerosis (MS) [1]. Although NLs may be present at any stage of the disease, widespread cortical demyelination is a typical feature of progressive MS [2]. According to the classification of NLs by Peterson and colleagues [3], subpial demyelinating NLs that spread from the pial surface with variable depth into the cortex, or type III NLs, constitute the most common manifestation of NLs observed with histopathology.

Presently, the identification of NLs in brains of MS patients using magnetic resonance imaging (MRI) is challenging both *in vivo* and *post mortem*. Limited resolution and contrast prevent current MRI methodologies from detecting the sub-millimeter pathological changes that arise with the occurrence of NLs.

The recent advent of ultra-high field MRI (7.0 Tesla [7T] and above) offers an opportunity to improve identification of NLs. Images at higher field strength have increased resolution, signal-to-noise ratio (SNR) and, for some applications, contrast-to-noise ratio (CNR). Within clinically feasible scan times of approximately 20 minutes, an SNR of about 10∶1 is available at an in plane resolution close to 200 microns (µm) using a 7T scanner and a multi echo gradient echo (ME-GRE) image acquisition technique [4]. T_2_^*^-weighted (T_2_^*^-w) ME-GRE images and reconstructed R_2_^*^ and phase maps at 7T may reveal detailed structures in various brain regions [5]–[8], including cortical laminae in grey matter (GM) as well as fiber bundles in white matter (WM) and cortex [6], [8].

At high field, contrast in T_2_^*^-w MRI is dominated by magnetic susceptibility effects, which can affect both the magnitude and the phase (through resonance frequency changes) of the signal, potentially providing new information about cortical pathology. Although the factors contributing to the magnetic susceptibility are not fully established [6], [9], [10], initial *post mortem* studies in MS brains have suggested that iron and myelin have dominant roles in lesion visibility [11]–[13]. Pathological and non-pathological areas of WM and GM of MS tissue show increased R_2_^*^ in regions with high iron content [11]–[13], which is mostly bound to ferritin or hemosiderin [11]. This iron may reflect the presence of healthy oligodendrocytes but also activated microglia and microbleeds [11], [13].

In this light we performed the present combined pathological and *post mortem* MRI study in order to [1] visualize NLs and [2] assess the sensitivity of 7T MRI in disclosing NLs relative to myelin and iron staining.

## Materials and Methods

### Study setting and ethics

This is a collaborative study between the National Institutes of Health in Bethesda, MD and the Medical University of Vienna, Austria. The study was bound by between-institutions material transfer agreements and complies with the declaration of Helsinki, the ethics requirement at the NIH and at the University of Vienna.

The two MS patients have expressed their wishes while alive of donating their bodies to a research institution for research studies and, upon patients' death - at outside institutions - their bodies were brought to the NIH for research purposes. In the light of patients' wishes while alive, the institutional review boards of both the NIH and the University of Vienna waived the need for additional consent. No Institutional IRB protocol was required to carry on the research procedures at the NIH. At the University of Vienna, the local ethics committee approved the work on pathological material (Ethics Committee Number 535/2004).

### Study design and material

**Figure 1:**
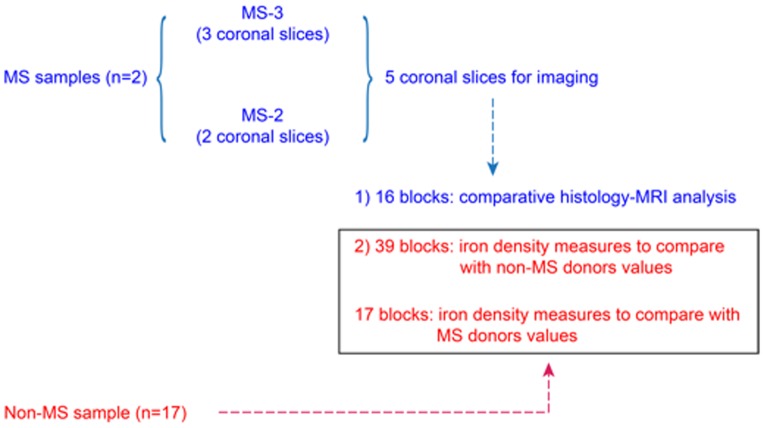
Study design.

Figure 1 depicts the study design. The study relies upon *post mortem* samples derived from two MS patients and 17 controls without neurological disease or brain lesions.

With respect to the MS samples, five 10 mm-thick coronal slices from the brains of two donors with secondary progressive (SP) MS, labelled MS1 and MS2, were analysed. These five slices were first imaged. Thereafter, 39 tissue blocks (i.e. 33 from MS1 and six from MS2) were derived. The tissue blocks of MS cases were chosen randomly without knowledge of the presence/extent of cortical pathology and were stained for myelin and iron content. These 39 blocks served to compare iron density between MS and non-MS samples. Of these 39 blocks, 16 blocks (i.e. 10 from MS1 and six from MS2) were chosen randomly and used for 7T MRI and comparative histology. With respect to the controls, 17 routine tissue blocks deriving from 17 individuals were utilized. No imaging data but only tissue sections stained for iron were generated with the brain specimens of controls. These tissue sections stained for iron served to compare iron density between MS and non-MS samples.

### Clinical data of the MS donors and controls

Detailed clinical features of donors have been previously reported [11] and are only briefly described here.

Sample MS1 derived from a Caucasian man whose MS onset occurred at age 40 with an episode of left optic neuritis. Between ages 42 and 51, the patient had 11 clinical relapses after which he progressed into a SP course. He developed permanent bladder symptoms and required a scooter at age 51. His clinical status subsequently deteriorated and by age 65, he was bedridden. He died of pneumonia at age of 70 in March 2005 after suffering from severe dementia for few a years. The patient was also afflicted by hypertension and atrial fibrillation. For his MS, the patient has never been treated with any disease modifying agents since the disease was most active during a, *era* when no disease modifying agents were available.

Sample MS2 derived from a Caucasian lady whose disease course was consistent with rapidly progressive MS. She presented with right optic neuritis at around the age of 37. Shortly thereafter she had an episode of bilateral hand paresthesias followed by an episode of gait disturbance. Her gait disturbance progressively worsened and by age 50, she was wheelchair-bound; by age 59, she needed assistance with daily activities. At the age of 61.5 a tracheostomy was done due to severe respiratory insufficiency and she died in February 2009 after developing pneumonia. This patient also suffered from mild hypothyroidism. During her lifetime the patient has been treated with several MS-therapies. These included: multiple courses of methylprednisolone, interferon beta (1a and 1b), intravenous immunoglobulins, intrathecal methotrexate and stem cell transplantation. She also had a baclofen pump to defeat spasticity.

The 17 control cases included in this study served as controls in prior studies [11], [14]. Exclusion of confounding pathology lesions has been performed using routine hematoxylin-eosin and Luxol fast blue periodic acid Schiff myelin stainings. Of these 17 controls, five were men and 12 were women. Average (± standard deviation) age was 60±23.3 years.

### Tissue collection and preparation for the MRI procedure and histopathology of MS samples

In each case, autopsy was performed within 24 hours of death. After autopsy each brain was immediately fixed in 4% neutral-buffered paraformaldehyde (PFA). For the MS1 case, whole-brain tissue was sectioned into 10 mm-thick coronal slices two weeks later. Due to the need of fresh tissue for unrelated studies in the case of MS2, the brain was cut immediately after death and prior to fixation. A few 10 mm-thick coronal slices were then fixed in 4% PFA for the imaging study. MRIs were performed at three (MS2) and 36 (MS1) months after death. Two slices from MS1 brain and three slices from MS2 brain were imaged.

### Image acquisition and reconstruction

Each brain slice was placed in a flat cylindrical, custom fabricated tissue container and imaged in 4% PFA [15]. Scans were acquired using a whole body GE Sigma 7T MRI scanner (GE Medical Systems) with a 24-channel receive-only array designed for imaging tissue slabs. A 3D T_2_^*^-w ME-GRE was performed using the following parameters: echo times (TEs)  = 8.7, 25.2, 41.7, and 58.2 ms; repetition time  = 200 ms; spatial resolution  = 0.21 mm isotropic, flip angle  = 20°, and bandwidth  = 62.5 kHz. Images were reconstructed using a phase-sensitive noise-weighted channel combination [16]. Quantitative R_2_^*^ maps were obtained using single exponential fitting to the images acquired at sequential TEs. Image reconstruction and calculation of R_2_^*^ were performed with program code created in-house using Interactive Data Language, version 7.0 (ITT Visual Information Solutions, White Plains, NY).

### Histology

Formalin-fixed brain tissue was cut into blocks measuring approximately 3×2×0.5 cm and embedded in paraffin. Serial 4 (3 to 5) µm thick sections were cut with a microtome and mounted on glass slides. For basic evaluation of general pathology and demyelination, hematoxylin-eosin and luxol fast-blue myelin stainings were performed in order to exclude confounding pathology. Immunohistochemistry (IHC) and iron histochemistry were performed as previously described [11]. For IHC, we used the avidin-biotin complex (ABC) method with diaminobenzidine (DAB) as chromogen. For antigen retrieval, sections were steamed in ethylenediaminetetraacetic acid (EDTA) buffer at pH 9 for 60 minutes in a household food steamer. The applied primary monoclonal mouse antibody against proteolipid protein (PLP, Serotec, product code MCA839G) was diluted 1∶1000. This antibody was applied twice, first overnight at 4°C and, after washing, a second time for 60 minutes at room temperature. A biotinylated secondary anti-mouse antibody (Jackson) was diluted 1∶500 and applied for 60 minutes at room temperature. For detection of non-heme tissue iron, the DAB-enhanced Turnbull blue staining was applied as previously described [11].

Sections stained for PLP were scanned with an Agfa Duoscan scanner at a resolution of 4000 pixels per inch. Images were stored as JPEG files, displayed on a screen and at the same time inspected with a Nikon Optiphot 2 microscope. Areas of demyelination were marked using Adobe Photoshop CS4. Complete WM demyelination was indicated with green. Complete GM demyelination was indicated with red. Areas of variably reduced myelin density in the GM (either incomplete demyelination or remyelination, which was not further determined) were indicated with yellow. Iron densitometry was performed on all slides stained for iron with a previously utilized method [14]. Images were taken with a Nikon DS-Fi1 digital camera mounted on a Reichert Polyvar 2 microscope using the software NIS elements (Nikon). For white balance, the auto-white function was applied. Identical brightness conditions were achieved by setting the intensity at 225/255 when no slide was under the objective. If possible, the center of each NL was matched with an image of adjacent NAGM. Images were saved as JPEG files. Image J 1.43r software was used to convert the colour images into 8-bit grey scale images. The mean grey value of each image was self-subtracted and a threshold was set at 60/255 to generate a black and white image capturing primarily intensely stained cell bodies and processes as well as iron-containing myelin sheaths. The percentage of black pixels was taken as a numerical estimation of iron staining intensity.

### NL classification

**Figure 2:**
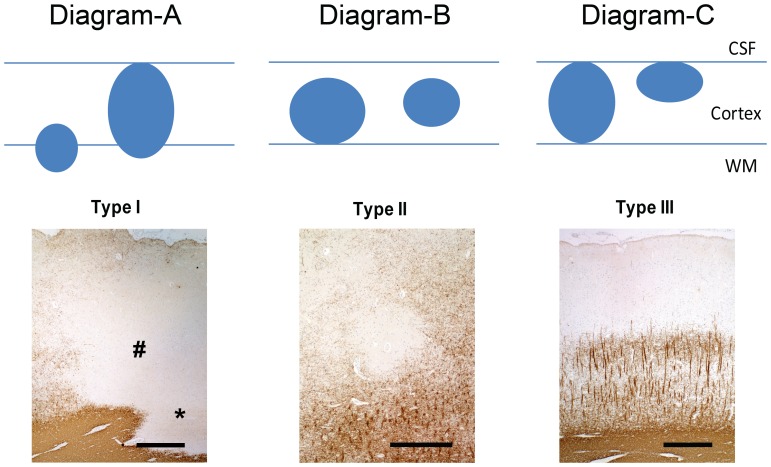
NL scheme and PLP staining. (**A**) Type-I NL with demyelination of the whole width of the cortex (#) and adjacent WM (*). (**B**) Type-II intracortical lesion evolving around a vessel. (**C**) Type-III subpial NL. Demyelination spreads from the pial surface until cortical layer 3. Scale bars represent 500 µm.

Following criteria from a previous pathological study [3], NLs were defined by both MRI and histology. *Type-I* NLs (leukocortical NLs) involved GM and WM (diagram-A in Fig. 2). *Type-II* NLs (intracortical NL) were NLs lying entirely in the cortical GM, without touching the pial surface (diagram-B in Fig. 2). *Type-III* NLs (subpial NLs) were NLs lying entirely in GM, touching the pial surface (diagram-C in Fig. 2).

### NL definition by MRI

MRI magnitude images and R_2_^*^ maps were used to identify and classify NLs. Once a NL was identified on one MRI slice, neighbouring MR slices were inspected carefully to ensure that signal changes were not due to artefacts or partial volume effects, and that a NL classified as Type-II and Type-III remained fully intracortical through each slice where identifiable. NLs were classified as areas of hypo- or hyper-intense signal with respect to the surrounding normal appearing GM (NAGM) using R_2_^*^/T_2_^*^ and magnitude images. The working hypothesis, based on previous studies on WM lesions [11], [12], was that NLs would appear bright on R_2_^*^ and dark on magnitude/T_2_^*^ images if iron accumulation was predominant. Conversely, NLs would appear bright on magnitude and dark on R_2_^*^ images if myelin and iron loss prevailed. In order to be counted, NLs had be visualized in all and each R_2_^*^, and magnitude/T_2_^*^ images.

### NL identification

NLs were identified by agreement of three investigators (FB, SH and BY) by first inspecting all MRI image slices. Additionally, MRI and histology stainings were inspected side-by-side to identify NLs in a subset of 16 tissue blocks. In these blocks, previously identified NLs were first identified by MRI, then by PLP-staining only (color-coded maps) and finally retrospectively assessed by MRI and PLP-staining side-by-side.

### Matching between MRI and histology

Images acquired from all tissue blocks stained for PLP and iron were stored as JPEG files. These images were then displayed alongside each corresponding 7T image for visual comparison.

### Statistics

NLs were counted prospectively (i.e., before the knowledge of their existence by histology) and retrospectively (i.e., with the knowledge of their existence by histology). Prospective MRI sensitivity (S_p_) was defined as TP_p_/(TP_p_ + FN_p_), where TP_p_ is the number of prospective true positive observations (i.e., NLs identified by MRI prospectively and confirmed by PLP staining) and FN_p_ is the number of false negative observations (i.e., sum of the NLs identified by MRI only upon PLP staining identification and the NLs seen by PLP staining only). Retrospective MRI sensitivity (S_R_) was defined as TP_R_/(TP_R_ + FN_R_) where TP_R_ is the total number of prospective and retrospective true positive observations (i.e., sum of NLs identified by MRI prospectively that were confirmed by PLP staining and NLs identified by MRI only upon PLP staining identification) and FN_R_ is the number of false negative observations (i.e., number of NLs seen only by PLP staining). Differences in iron content between areas of NLs and NAGM were assessed using the Mann-Whitney *U* test (pooled data of MS cases) and Wilcoxon signed ranks test for related samples (separate analysis of the two MS cases).

## Results

### Image inspection

**Figure 3:**
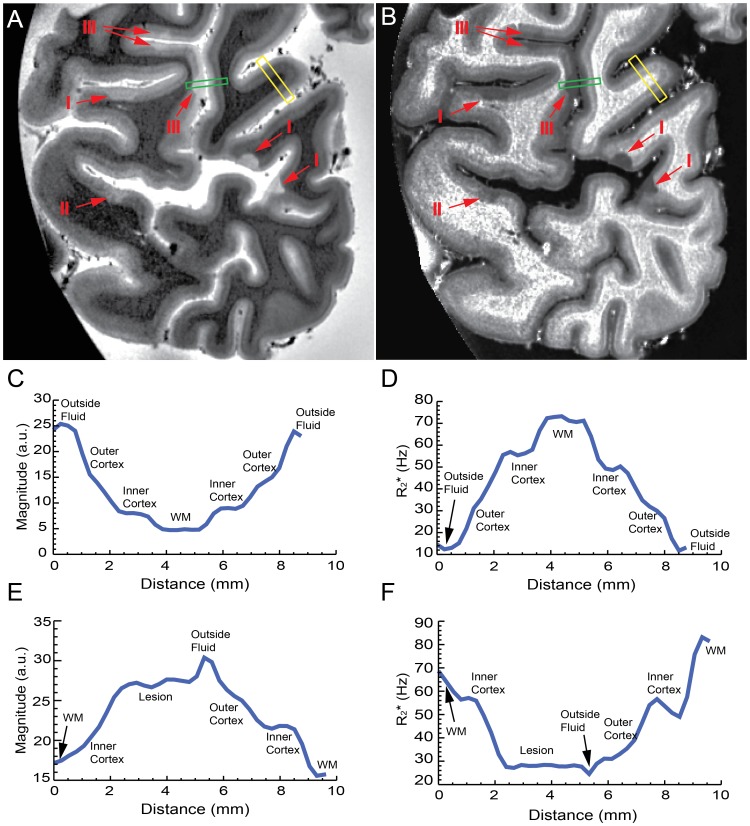
MRI contrast. Examples of NLs from tissue MS2 identified on MRI (**A**) magnitude (TE = 25.2 ms), and (**B**) R_2_^*^ images. Arrows point towards type-I, type-II and type-III NLs. Examples of intensity profile on of cortical regions with (green box) and without (yellow box) NLs on both magnitude (**C and E**) and R_2_^*^ maps (**D and F**). In this example, the WM has a R_2_^*^ value of around 75 Hz, while the inner part of cortex has a R_2_^*^ of around 45 Hz, the outer part of cortex is around 40 Hz and NL is as low as around 30.

Excellent MRI contrast was achieved in both magnitude and R_2_^*/^/T_2_^*^ maps, which allowed discerning without ambiguity the boundaries between the GM and WM. The high MRI resolution also allowed for the distinguishing of some cortical layers. Figure 3 shows several types of NLs seen on MRI scans of one tissue sample from patient MS2.

The magnitude image showed a progressive darkening from superficial to deep layers of the cortex (Fig. 3A). The opposite was seen in the R_2_^*^ maps (Fig. 3B). An intensity profile on magnitude (Fig. 3C) and R2* maps (Fig. 3D) demonstrates the laminar MRI contrast along a selected region indicated by the yellow box in Figure 3B. Multiple inflection points in the profile curve corresponding to the distance range within cortex indicate multiple cortical layers in MRI magnitude and R2* maps. Another intensity profile across a NL (indicated by the green box in Figure 3B) and covering neighbouring parts of WM, GM, NL and fluid bath (Fig. 3E and Fig. 3F) is also delineated and shown on both magnitude and R_2_^*^ maps.

### NL occurrence by MRI

NLs were visible on both MRI magnitude and R_2_^*^ maps of the five imaged brain slices obtained from the two patients. Categorization into the different NL types depending upon their location in the cortex was possible (Fig. 3A and Fig. 3B). All identified NLs appeared darker in R_2_^*^ images and brighter in magnitude images with respect to the surrounding NAGM. No other types of signal changes were seen.

MRI identified a total of 93 NLs in the five slices from the two brains. NL types on the basis of MRI appearance are listed in Table 1. Subpial NLs made up smaller proportions of NLs in both samples. However, while the majority of NLs identified in MS1 were Type-I NL, an equal distribution between Type-I and Type-II NLs was seen in MS2.

**Table 1:** NL Occurrence.

NL OCCURRENCE BY MRI (5 brain slices)				
	Type-I NLs	Type-II NLs	Type-III NLs	Total
MS1	29 (60.4%)*	17 (35.4%)*	2 (4.2%)*	48
MS2	16 (35.6%)**	20 (44.4%)**	9 (20%)**	45
**Total**	**45 (48.4%)** ***	**37 (39.8%)** ***	**11 (11.8%)** ***	**93**

*% of 48 total number.

**% of 45 total number.

***% of the total number 93.

### MRI sensitivity in detecting NLs

For a subset of 16 tissue blocks, we analysed NL conspicuity on MRI and PLP stainings side-by-side. In these, a total of 65 NLs were identified, 55 in MS1 and 10 in MS2. Of these 65 NLs, 30 (46%) NLs were identified prospectively by MRI (TP_p_), 33 (51%) NLs were seen only by PLP staining (FN_p_) and two (3%) NLs were seen only by MRI (prospectively false positive, FP_p_). Twelve (18%) NLs were seen by MRI only after being identified by PLP staining (TP_R_), totalling 42 (64%) NLs seen by MRI (TP_r_); 21 (32%) NLs remained seen only by PLP staining (FN_R_) and two (3%) NLs were seen only by MRI (FP_p_ and FP_r_). Compared to PLP staining, MRI had a S_p_ and S_r_ of 48% and 67%, respectively.

### NL appearance by MRI and PLP staining and MRI

**Figure 4:**
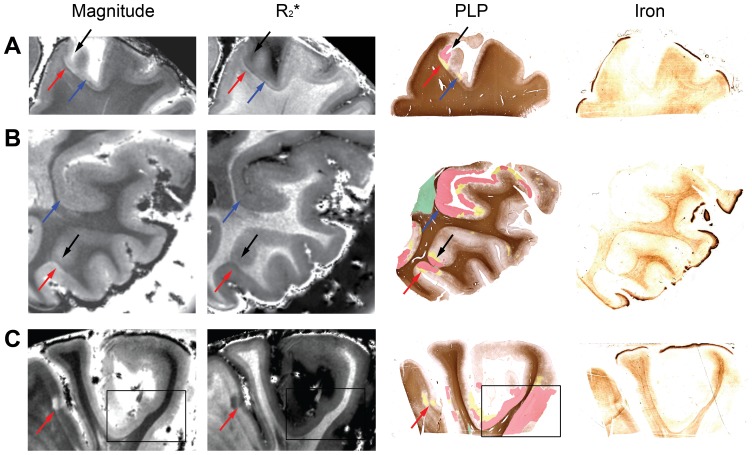
NLs on MRI and tissue sections stained for PLP and iron. Examples Examples of NLs from tissue MS1 (**A, B and C**) identified on MRI magnitude (TE = 25.2 ms) and R_2_^*^ images as well as color-coded PLP-staining and iron staining. Red arrows point towards NLs identified by MRI and confirmed to correspond to area of demyelination by the color-coded PLP staining. Blue arrows point towards NLs identified by the color-coded PLP staining and only retrospectively identified by MRI. Black arrows and black box point towards NLs identified by the color-coded PLP staining and not by MRI even upon a second retrospective image inspection. In the black box we include a large area of demyelination which goes entirely undetected by MRI. In the color-coded PLP staining of figure: green  =  complete WM demyelination, red  =  complete GM demyelination, yellow  =  areas of variably reduced myelin density.

Twenty NLs not necessarily identified by MRI (either prospectively or retrospectively) as focal and apparently circumscribed type-I or type-II NLs were found to be incompletely detected areas of extensive demyelination in the PLP staining, extending from the subpial surface across the entire cortical depth and spreading tangentially to the surface over one or more gyri. Examples of these NLs are displayed in Figure 4. In Figure 4A to 4C, NLs were identified by both magnitude and R_2_^*^ MRI prospectively (red arrows) and retrospectively (blue arrows). The lesions were confirmed by the PLP staining as indicated in red or yellow (see legend for more detail). Nevertheless, the PLP staining also showed extensive demyelination, which was only barely visible by MRI, as indicated by the black arrows in Figure 4A and 4B. Similarly, in Figure 4C there is extensive demyelination, framed in line rectangles, which is not apparent from MRI.

### NL appearance by MRI and iron staining and densitometry values

**Figure 5:**
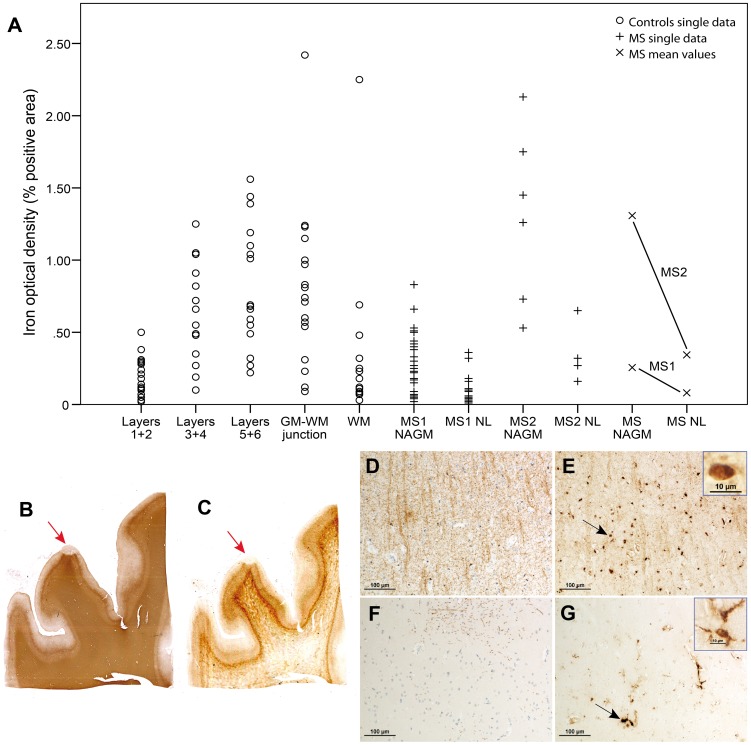
Iron loss in NLs. Iron densities from different layers of the 17 control cortices are shown together with densities from MS1 and MS2, which are displayed separately and pooled together (mean values) (**A**). See text for statistical analysis. MS1 showed lower global iron values than MS2, which is likely due to formalin fixation time. PLP myelin staining (**B**) and iron staining (**C**) on two consecutive slides of tissue MS2 disclose iron loss together with myelin loss in a NL (red arrows). Higher magnification (**D,** **E, F, G**) of PLP (**D, F**) and iron stainings (**E, G**). In NAGM (**D**, **E**), iron is present in oligodendrocytes (black arrow and inset in **E**) and myelin sheaths. In a NL (**F, G**), iron is removed from the demyelinated cortical parenchyma and present in perivascular macrophages and microglia (black arrow and inset in **G**).

For the same 16 tissue blocks, we also compared MRI and iron stainings side-by-side. R_2_^*^ increases were not observed by qualitative inspection. As exemplified in the iron stainings in Figure 5, iron was invariably reduced in NLs. Quantitative analyses by digital optical densitometry were performed in several regions of layers 3+4 NAGM (n = 33 for MS1 and 6 for MS2) and layers 3+4 NLs (n = 26 for MS1 and 5 for MS2) in MS patients as well as one region per different cortical layers in each one of the 17 samples derived from the controls (Fig. 5).

For each MS sample we had several measurements that derived from two samples only, whilst for controls we had fewer individual measurements deriving from a larger cohort. Such discrepancy in number of samples, i.e., 17 controls *vs* 2 MS cases precluded performing statistical analysis between groups. Therefore, we provide only a descriptive analysis. Yet, from such a description we can visualize an expected gradient of iron density in healthy cortex depending upon the anatomical layer as well as the marked difference between healthy cortex and NLs. Such a difference in iron content is also reflected in the gradient of signal intensity on R_2_^*^ seen in Figure 2.

Statistical analyses were performed to compute differences in iron content between 31 NLs areas and 39 NAGM all derived from MS patients. The results of such an analysis showed iron density to be lower in the demyelinated NLs than in the adjacent NAGM (Fig. 5A), partly mirroring the myelin loss indicated by PLP staining (Fig. 5B and 5C).

Differences in iron density between NLs and NAGM were significant when the two brains were analysed separately (p = 0.001 for MS1 and *p* = 0.043 for MS2) and when pooled together (p = 0.001). Figure 5D to 5G shows PLP and iron stainings of NAGM and a NL at higher magnification, where the removal of iron from demyelinated cortical parenchyma is depicted.

## Discussion

Our data represent a step forward from previously reported findings at lower field strength where sensitivity of MRI in depicting NLs was much lower compared to histopathology [17], [18]. However, compared to previous studies at ultra-high field MRI, we report a relatively low retrospective MRI sensitivity. In a study performed using T_2_-w MRI at 9.4T, 78% of pathologically detected NLs were seen retrospectively [19]. Subsequently, Pitt and collaborators reported that 3D T_2_^*^-w GRE and WM-attenuated inversion recovery turbo-field-echo (TFE) sequences at 7T prospectively detected 46% (T2*) and 42% (TFE) NLs, which is similar to our 48% of prospective sensitivity [13]. Retrospectively however, they detected 93% and 82% of all NLs, whereas here we report 67%. Pitt and collaborators [13] used very similar imaging techniques and identical MRI field strength, at a somewhat lower image resolution. One could argue that identifying NLs by a positive signal change (i.e., increase signal in T_2_^*^-w images) rather than by a negative signal change in a quantitative MR image (i.e., decrease signal in R_2_^*^ maps) as done in our work could have permitted an easier NL identification. Against this argument is the fact that as done in our study, also Pitt and collaborators used magnitude images, where NLs are indeed visible as positive signal change. Another possible explanation of this inter-study variability is the use of color-coded PLP maps, which enhanced the visibility of the extent of demyelination with respect to the PLP stainings alone. It is also conceivable that the differences in histopathological characteristics of the samples we studied could account for some of the discrepancies between our findings and the ones by Pitt and collaborators. The latter authors examined the brains of three MS cases in the progressive stage of the disease. Rings of activated iron-laden microglia were observed around some NLs, suggestive of active demyelination at the lesion edges. Conversely, all NLs in our samples were devoid of iron-positive lesion edges and inactive, the latter being typical of chronic progressive disease. The inter-study variability goes along with the findings by Kooi and co-authors who have shown that rims of activated microglia at the border of the NLs may not always be present in brains of MS patients, rather in a subset of patients with more active WM inflammation [20].

We found non-subpial NLs to be the predominant type of NLs disclosed by MRI in each sample. Conversely, a detailed analysis of NL occurrence by histopathology showed extensive subpial demyelination to frequently occur despite its lack of visibility by MRI. The results parallel and explain the discrepancy between *in vivo* and *post mortem* findings. With only one exception [21], *in vivo* studies at different magnetic fields with the use of sequences exploiting different contrast mechanisms support type-I and type-II NLs to be more easily observed in MS patients [22]–[28]. The notion contradicts *post mortem* findings, where subpial NLs predominate [2], [29]. Our results demonstrate that the technique, i.e. MRI, sets the limit for NL visibility and subpial cortical pathology is indeed more extensive than what imaging can disclose.

In contrast to previous observations [30], our observations indicate that lesion size may not necessarily contribute to the difference in ability of histology and MRI to detect lesions. For several large subpial NLs, we observed MRI maps successfully identifying only a small portion. These findings pertained to NLs with features either of complete/incomplete demyelination or remyelination. Several factors may have affected the current findings. First, lesion detection relies to some extent on the local laminar contrast, which varies considerably among cortical regions [4], [31], partly reflecting the underlying function. This factor likely complicates the detection of large NLs, as they can span several functional areas with varying laminar patterns. Secondly, although myelin may be the dominant source of contrast in ME-GRE images [32], it does not exclusively contribute to it. The presence of other components such as phospholipid debridement [33] may counteract the effect of myelin loss on MRI. Third, in this study we show reduced iron content in inactive NLs of two MS cases, similar to the observed iron loss in inactive WM lesions [14] and deep GM lesions [34]. In the normal cortex, iron within ferritin is found predominantly in oligodendrocytes and myelin sheaths [4]. When these structures are destroyed in the course of demyelination, iron is taken up and presumably removed by microglia and macrophages [14], [35]. The resulting iron loss in NLs might augment the signal change of myelin loss in NLs, which could hold especially true for cortex with high constitutive iron load, such as the primary motor cortex [36]. Last, focal NLs might be more easily detected when the surrounding GM is healthy, thus preserving the necessary MRI contrast. Newly forming inflammatory NLs, which present with an iron rim, might be readily detected in patients with short disease duration or low overall cortical involvement. However, because of the lack of contrast between healthy and diseased GM, MRI may underestimate especially the presence of large cortical involvement with inactive lesions in patients with long-standing disease.

Some limitations of our work need to be considered before drawing conclusions from our study. First, the small number of examined samples requires confirmation with larger studies. The small sample size precluded statistical analysis assessing quantitative measurements between healthy and diseased brains and limit the value of some our findings as merely descriptive. Secondly, although the time between death and formalin fixation of the samples is comparable between the two brains, the time between fixation and imaging was different. Such difference introduces some confounders. From an MRI stand point it is well known that formaldehyde fixation facilitates protein cross-linking [37] thereby reducing the T2 relaxation time [38]. Overall *post mortem* samples have reduced T2 values compared to *in vivo* tissues [39]. Since we did not base our results on MRI-derived quantitative measures, we believe that this factor may not necessarily translate into a drawback for our study. In addition, possible differences in the degree of iron leak [40] need to be considered. Last, lack of information regarding axonal and neuronal density precludes the understanding of their role in explaining differences in signal detection by MRI associated to NLs.

Despite these limitations, the work presented here provides an indication that the detection of MS-induced NLs with high field MRI should precede with caution. Although small in size, our sample is sufficient to demonstrate that the sensitivity of MRI even at ultra-high field coupled with surface coil and ultra-high resolution techniques, essentially unfeasible *in vivo*, remains limited.
